# Neuropeptide S and BDNF gene expression in the amygdala are influenced by social decision-making under stress

**DOI:** 10.3389/fnbeh.2014.00121

**Published:** 2014-04-08

**Authors:** Justin P. Smith, Justin K. Achua, Tangi R. Summers, Patrick J. Ronan, Cliff H. Summers

**Affiliations:** ^1^Department of Biology, University of South DakotaVermillion, SD, USA; ^2^Neuroscience Group, Division of Basic Biomedical Sciences, Sanford School of Medicine, University of South DakotaVermillion, SD, USA; ^3^Research Service, Sioux Falls VA Healthcare SystemSioux Falls, SD, USA; ^4^Avera Research Institiute, Avera McKennan Hospital and University Health CenterSioux Falls, SD, USA

**Keywords:** stress, decision-making, neuropeptide S, BDNF, escape, submission, aggression

## Abstract

In a newly developed conceptual model of stressful social decision-making, the Stress-Alternatives Model (SAM; used for the 1st time in mice) elicits two types of response: escape or remain submissively. Daily (4d) aggressive social interaction in a neutral arena between a C57BL6/N test mouse and a larger, novel aggressive CD1 mouse, begin after an audible tone (conditioned stimulus; CS). Although escape holes (only large enough for smaller test animals) are available, and the aggressor is unremittingly antagonistic, only half of the mice tested utilize the possibility of escape. During training, for mice that choose to leave the arena and social interaction, latency to escape dramatically decreases over time; this is also true for control C57BL6/N mice which experienced no aggression. Therefore, the open field of the SAM apparatus is intrinsically anxiogenic. It also means that submission to the aggressor is chosen despite this anxiety and the high intensity of the aggressive attacks and defeat. While both groups that received aggression displayed stress responsiveness, corticosterone levels were significantly higher in animals that chose submissive coexistence. Although both escaping and non-escaping groups of animals experienced aggression and defeat, submissive animals also exhibited classic fear conditioning, freezing in response to the CS alone, while escaping animals did not. In the basolateral amygdala (BLA), gene expression of brain-derived neurotrophic factor (BDNF) was diminished, at the same time neuropeptide S (NPS) expression was significantly elevated, but only in submissive animals. This increase in submission-evoked NPS mRNA expression was greatest in the central amygdala (CeA), which coincided with decreased BDNF expression. Reduced expression of BDNF was only found in submissive animals that also exhibit elevated NPS expression, despite elevated corticosterone in all socially interacting animals. The results suggest an interwoven relationship, linked by social context, between amygdalar BDNF, NPS and plasma corticosterone.

## Introduction

Social stress is the most potent type of stressor (Koolhaas et al., 1997). It is a product of fear-learning and anxiety derived from the unpredictable and uncontrollable nature of an aggressive conspecific interaction (Koolhaas et al., 1997, 1998; Summers et al., 2005a). Elements of stress and reward-related circuitries, that include amygdala, prefrontal cortex (PFC), cingulate cortex, nucleus accumbens, ventral tegmental area, hippocampus, paraventricular hypothalamus, are responsible for resilience or susceptibility to stress and emotional disorders, producing adaptive social responses such as submissive, aggressive, or escape behaviors (Krishnan et al., 2007; Feder et al., 2009; Arendt et al., 2012; Tanaka et al., 2012). Decision-making processes are influenced by stressors, because they involve neurocircuitry that includes emotional and executive brain regions (Bechara et al., 1999; Brand et al., 2007; de Visser et al., 2010, 2011a,b). Decision-making circuitry includes the amygdala, orbitofrontal cortex, anterior cingulate, dorsolateral PFC, ventral and dorsal striatum (Bechara et al., 2003; de Visser et al., 2011a; Koot et al., 2012). This suggests that stress-related circuitry that includes the amygdala may be important for social decision-making (Carpenter and Summers, 2009; Arendt et al., 2012). While submissive, aggressive, or avoidance responses are considered elements of anxious and depressive disorders in human populations, they are also adaptive reactions to environmental and social stressors, and thereby important factors in decisions regarding social and environmental conditions.

Decision-making includes neural plasticity such as long-term potentiation, synaptic remodeling, potentially resulting in enhanced learning for which the mechanisms likely include brain-derived neurotrophic factor (BDNF), its receptor tropomyosin related kinase B (TrK_B_) and AMPA receptor subunits including GluR_1_ or GluR_4_ (Broad et al., 2002; Gasic et al., 2009; Kang et al., 2010; da Rocha et al., 2011; Diógenes et al., 2011). Decisions influenced by social stress, fear or anxiety are responses modified by experience (learning). The neuroplastic changes accompanying decision-making involve modulation by the basolateral amygdala (BLA) and related circuitry (Davis, 1980, 2006; LeDoux et al., 1990; Broad et al., 2002; Monfils et al., 2007; Fanous et al., 2010; Lonsdorf et al., 2010; Razzoli et al., 2011; Roth et al., 2011; Orsini and Maren, 2012). Additionally, amygdalar BDNF and TrK_B_ stimulates acquisition of social defeat conditioning in hamsters (Taylor et al., 2011). Heightened anticipatory stress responses, such as enhanced plasma cortisol, along with increased susceptibility to stress-induced affective disorders in humans are linked to a variant (Val66Met; G196A) of the BDNF gene (Schenkel et al., 2010; Colzato et al., 2011) and influence more emotionally constrained decision-making (Gasic et al., 2009; Kang et al., 2010; da Rocha et al., 2011). Motivational aspects of social interaction and social aversion require increased and inhibited expression respectively, of the gene for BDNF (Berton et al., 2006). Other neuropeptides are also responsible for modifying the activity of neural circuits related to motivation, such as neuropeptide S (NPS) and NPY, which influence arousal, stress, reward, memory, and reduce anxiety (Morgan et al., 2000; Guerrini et al., 2010; Tasan et al., 2010; Jungling et al., 2012; Cannella et al., 2013). Produced in the brainstem, NPS has terminal fields in regions associated with stress, anxiety, and fear learning, such as amygdala and piriform cortex (Xu et al., 2004, 2007; Jungling et al., 2008; Meis et al., 2008; Guerrini et al., 2010). Stress and accompanying corticosteroids, inhibit amygdalar BDNF expression (Pizarro et al., 2004), which may result in social avoidance (Berton et al., 2006). Considering the involvement of NPS or NPY in modifying circuits related to motivation, these anxiolytic peptides may be affected as well. While BDNF modifies learning and plasticity (Broad et al., 2002; Diógenes et al., 2011), NPS decreases anxious behavior (Jungling et al., 2008; Dannlowski et al., 2011; Ruzza et al., 2012; Wegener et al., 2012) while concurrently increasing arousal (Guerrini et al., 2010; Ionescu et al., 2012). Together the unique pro-arousal anxiolytic attributes of NPS and neuroplastic qualities of BDNF suggest the potential for a synergistic role in contextually derived adaptive behavior during decision-making.

These experiments were designed to discover whether stressful decision-making influences gene expression related to neuroplasticity (BDNF, TrK_B_, GluR_1_, GluR_4_) and anxiety (NPS, NPY) in a brain region known to participate in decision-making. We predict that decisions made under social stress will alter expression of these signaling molecules and receptors in the amygdala. To test this idea we have developed a model (Stress-Alternatives Model (SAM)) and behavioral arena that allows for a choice of responses during social defeat: (1) escape, via one of two escape routes; or (2) remain submissively (Arendt et al., 2009, 2012; Carpenter et al., 2009a). First, we hypothesized that exposure to an aggressive social interaction (as in the SAM) will reveal inherent behavioral differences within the original population, such that two clear cut groups emerge: those exhibiting escape behavior and others exhibiting submissive behavior. Secondly, we hypothesized that escape behavior will be influenced by social and environmental learning, resulting in decreased latency to escape. We also hypothesized that submissive animals will exhibit classical fear conditioning (Carpenter and Summers, 2009). In addition, we hypothesized that aggressive social interaction in the SAM will increase plasma corticosterone, and do so significantly more in submissive animals (Carpenter and Summers, 2009). As stress and glucocorticoids have been shown to inhibit BDNF expression, and social stress has been demonstrated to inhibit BDNF expression the BLA, we hypothesized that submissive animals would exhibit decreased BLA BDNF and TrK_B_ gene expression (Pizarro et al., 2004). In addition, as the BLA projects to the central amygdala (CeA), we hypothesized that BDNF and TrK_B_ gene expression would also be inhibited in the CeA by social submission in the SAM. We further hypothesized that elevation of the stress hormone corticosterone would be alleviated by learning to escape, and that BDNF and TrK_B_ gene expression would therefore not be inhibited. We also examined receptor subunits of the glutamatergic AMPA receptor that are correlated in amygdala with increased latency to escape in hamsters (Arendt et al., 2012). We hypothesized that, like hamsters and trout, mice would exhibit elevated GluR_1_ (GluA1) and also GluR_4_ (GluA4), following social aggression and submission (Carpenter et al., 2009b; Arendt et al., 2012). Finally, we hypothesized that expression of NPS and NPY in the BLA and CeA will increase in animals that experience social aggression, and become especially elevated in animals that do not learn the escape behavior.

## Materials and methods

### Animals

**Figure 1:**
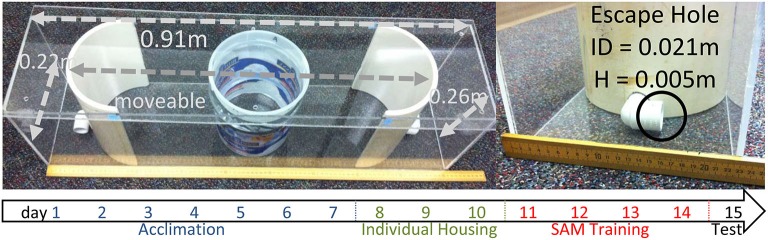
**SAM apparatus**. The behavioral apparatus for the SAM social interactions is 0.91 × 0.22 × 0.26 m with an internal arena for social interactions that can be adjusted, but was set at 0.70 m for these experiments. No corners exist in the interaction arena, however there are two escape routes placed at opposite ends of the apical extent of the behavioral arena. The escape routes are 0.021 m in internal diameter, with the lower rim of the hole 0.005 m above the floor. A large CD1 aggressor is placed outside the opaque cylinder, and then a test animal is placed inside an opaque cylinder 30 s prior to social interaction. Below: Time line indicating events (blue: group housing, cage acclimation; green: individual housing; red: social interaction in SAM apparatus; black: testing without aggressor, sampling) for test animals by day.

Adult (8 weeks) male C57BL6/N mice weighing ∼23 g (Harlan, Indianapolis; *N* = 50) were group housed, four animals per cage, during a 7 day acclimation, and then housed singly for the duration of the experiment (8 days; Figure 1). A separate cohort of animals, retired Hsd:ICR (CD1) male breeders weighing ∼53 g were used to provide aggression during the behavioral portion of the experiment (Harlan, Indianapolis; *N* = 15). Mice were on a 12:12 reversed light-dark cycle (lights off at 10 AM) with food and water provided *ad libitum*. Behavioral testing took place between 10 AM and 4 PM. All experiments were executed in a manner that minimized suffering and the number of animals used, in accordance with the National Institutes of Health’s Guide for the Care and Use of Laboratory Animals (NIH Publications No. 80–23), and approved by the Institutional Animal Care and Use Committee of the University of South Dakota.

### Experimental design

The SAM behavioral apparatus is constructed using 5 mm thick transparent acrylic sheets formed to make a rectangular box (L:91 cm, W:22 cm, H:26 cm) with a lid (L:91 cm, W:25 cm; Figure 1). Inside the SAM box are two semicircular polyvinyl chloride sections with a 21 mm diameter hole 5 mm off the bottom portion of each section (escape routes). The holes are constructed from 3/4 inch diameter, 90° polyvinyl chloride plumbing tubes. Each semicircular polyvinyl chloride section was placed inside the rectangular box with the holes 70 cm apart creating an oval area and a space outside the oval portion between the semicircular polyvinyl chloride section (Diameter: 22 cm, H: 26 cm) and the edge of the rectangle box. The positioning of the semicircular end-pieces (each containing an escape hole) is adjustable to increase or decrease the available area of open field exposure. In addition, a removable opaque cylinder (diameter 16.5 cm, height 22 cm) is placed in the center of the oval open field area to separate the test animal from the aggressor. A 5 mm thick transparent acrylic sheet lid measuring 25 cm wide by 91 cm long is placed over the box to ensure animals not jump out.

### Pre- and during sam analysis of potential anxious phenotypes

In SAM experiments, test animals are subject to aggression (see Section Social Interaction/Behavioral Procedures) and respond either by escaping (*N* = 20) or remaining submissively (*N* = 22). Based on *a priori* hypotheses, preliminary experiments, and previous results, we used the population of escaping mice, and the population of submissive mice to constitute separate experimental groups. To examine whether these self-selected groups (and controls) were predetermined by innately anxious behavior, a group of mice were tested on the elevated plus maze (EPM; *N* = 43). Animals which were eventually classified as belonging to groups of escaping (*N* = 12) submissive (*N* = 17), or control (*N* = 14) animals, were analyzed for time in open and closed arms of the EPM as measures of adventurous or anxious behavior, respectively. During the SAM experiments, two additional control groups were examined for comparison: animals left undisturbed in normal housing (*N* = 12) and animals subject to the SAM apparatus without aggression but with CS (see below) on training and test days (*N* = 8).

During SAM experiments time spent in the center of the oval arena of the SAM was measured to examine open-field anxiety. Anxious behavior was measured by time spent along the edges, avoiding the open center area. This open field test during SAM experiments differs from standard open field tests, because of three unique qualities of SAM experimentation: (1) escape holes are available; (2) a large CD1 aggressor may be present; and (3) comparisons are made among groups, regardless if there was an aggressor present or if escape was accomplished. As time spent in the SAM apparatus was different between escaping and non-escaping (submissive) groups, data were normalized to percentage of total time spent in the arena. Additional animals were included in the open-field test that were not included for analysis in other behavioral and molecular assessments (No Aggression; *N* = 7, Escape; *N* = 11, and Submission; *N* = 32).

### Social interaction/behavioral procedures

Behavioral observations were manually and digitally recorded. The CD1 aggressor was placed into the SAM inside the oval area but outside the opaque cylindrical divider. A C57BL6/N test mouse was placed inside the cylindrical divider and allowed 30 s to acclimate. All training days (1–4) as well as the test day (5) were run in the SAM apparatus (1 trial/animal/day). All trials for the No Aggression group took place in the SAM apparatus with no CD1 present but with access to escape holes each day. On training days, after test or control animals were in place, a tone (2500 Hz at 75 dB) was sounded for 15 s, followed by 15 s of silence. After the silence, the opaque cylinder separating the two animals was removed (presentation of the unconditioned stimulus (US)) allowing the animals to interact for a maximum of 300 s; submissive animals remained for the entire 300 s with the CD1. The time allowed for social interaction minimized injury to the test mouse, because the average latency to attack was ∼30 s, with an average of four attacks per interaction. Attacks were defined as a successful bite by the CD1 on the test animal. A novel CD1 is used for each interaction (used once per day), to limit habituation; mice often display more interest in novel, compared to familiar, conspecifics (Young, 2002; Toth and Neumann, 2013). The duration of the interactions varied, because some animals escaped and some did not, and among those that did, there were also differences in individual escape latency.

On test day, test mice were placed in the SAM apparatus as on training days, with the exception that no CD1 aggressor was present. The CS was given, and if applicable, latency to escape recorded. Animals were removed from the SAM apparatus after 300 s and brain and blood samples collected.

Latency to escape was measured from removal of the cylinder, which allows for presentation of the US (aggressor), to the moment at which the animal passed through the escape portal. Duration of interaction was defined as the period from lifting of the cylinder to the moment that the animal exited, using one of the two available escape holes, or 300 s of interaction for submissive animals. Interactions were scored (two naïve independent trained scorers) for attacks made and latency to escape. The tone served as a conditioned stimulus (CS), while aggression from the larger animal was the US. If the test animal utilized an escape hole during the experiment a cover was placed over the hole for the remainder of the allotted 300 s. In addition to the animals that had an opportunity to escape from social aggression, there were two control groups of C57BL6/N mice. One group was exposed to the SAM apparatus with no CD1 present but identical in all other aspects. The other control group consisted of animals that remained individually housed in their home cages for the duration of the experiment.

### Hormonal analysis

Ten minutes after behavioral testing on day 5, animals were decapitated; trunk blood was centrifuged for 2 min, brains were collected and frozen at −80°C. Plasma corticosterone concentrations were quantified in duplicate using a corticosterone enzyme-linked immunosorbent assay kit (Enzo Life Sciences, Farmingdale, NY).

### Quantitative rtPCR

Frozen brains were sliced coronally (200 µm), and from the amygdala the BLA and CeA were microdissected on a freezing block (−30°C) using coordinates (BLA −0.58 to −1.06 mm; CeA −0.70 to −1.06 from Bregma) from a mouse brain atlas (Paxinos and Franklin, 2004), with the blunt tip of a 23 gauge needle (Palkovits, 1973). Samples were immediately injected into lysis buffer (RNAqueous-Micro Kit, Life Technologies Corp.) before homogenization with a pestle. Total RNA was extracted from microdissected samples using RNAqueous-Micro kit (Life Technologies Corp.) and quantified using Agilent 2100 Bioanalyzer (Agilent Technologies, Santa Clara, CA). Purified RNA was then used for complementary DNA (cDNA) synthesis in 20 µl reactions using the High Capacity cDNA archive kit (Life Technologies Corp.). For all qPCR reactions 2 µl of total cDNA product was used in 20 µl reactions. Step One Plus Real-Time PCR System (Life Technologies Corp.) was used to perform all qPCR reactions using Taq-man Assay On Demand primer/probe sets (Life Technologies Corp.) for Glyceraldehyde 3-phosphate dehydrogenase (GAPDH; Mm99999915_g1), BDNF (Mm04230607_s1), TrK_B_ (Mm00435422_m1), NPS (Mm03990645_m1), NPY (Mm03048253_m1), GluR_1_ (Mm00433753_m1) and GluR_4_ (Mm00444754_m1). The primer/probe set used for BDNF covers 11 BDNF reference sequences and both primers and probes map within each exon. Each sample was run in duplicate and normalized to the expression of housekeeping gene, GAPDH. The TaqMan qPCR was performed at 50°C for 2 min and 95°C for 10 min, followed by 50 cycles at 95°C for 15 s and 60°C for 1 min. The animals in each group were considered biological replicates, and changes in gene expression were either represented individually (regressions) or averaged (group means). The qPCR reactions for each animal were repeated twice and results from individual reactions were averaged. Changes in gene expression were quantified by real-time qPCR and analyzed using the 2^−∆∆CT^ method (Livak and Schmittgen, 2001), comparing all samples to the average ∆C_T_ value of the control animals (not exposed to the SAM apparatus). Values for qPCR data were expressed as mean fold change ± standard error of the mean (SEM).

### Statisitical analysis

Plasma corticosterone, gene expression, and open field results were compared across groups (Escape, Submission, No Aggression and Cage control) using one-way ANOVA. Comparison of time spent in the center and the edges of the open arena were compared by paired *t*-tests. It is important to note that additional animals were included in the open-field tests that were not included for analysis in other behavioral and molecular assessments. Further, during behavioral analyses or qPCR low cDNA quantity or lost tissue/samples resulted in some data being omitted from analyses. Significant effects between groups for one-way analyses were examined with Student–Newman– Keuls *post-hoc* analyses (to minimize Type I error) and Duncan’s Multiple Range Test (to minimize Type II error). Behavioral comparisons across days were made using one-way repeated measures analyses followed by Holm-Sidak method *post-hoc* tests. To assess the relationships between individual corticosterone concentrations with BDNF and NPS gene expression in BLA and CeA we used multiple regression and 3D regression analyses.

## Results

### Social behavior

All C57BL6/N exposed to the SAM quickly investigated the escape holes and the novel CD1 (when present) following the removal of the cylinder divider at the beginning of each interaction. During training with a larger aggressive conspecific, test mice were attacked an average of four times beginning at ∼30 s after exposure to the CD1. About half of the test mice escaped and half remained submissively, similar to what we have previously seen with other species (Arendt et al., 2009; Carpenter and Summers, 2009). It is important to note that of the approximately 50% submissive mice 7% escaped initially before choosing submission and 9% of the escaping mice remained submissive during the initial interaction before choosing to escape for the remainder of the experiment.

### Pre-sam analysis of potential anxious phenotypes

The C57BL6/N mice that eventually demonstrated self-selected escape or submissive behaviors, as well as a group of control animals, were tested on the EPM to discover whether these eventual groups were innately disposed to anxious behavior. There were no significant differences among any groups on the time spent in the open arms (*F*_2,40_ = 0.0097, *p* < 0.99; data not shown) or in the closed arms (*F*_2,40_ = 0.34, *p* < 0.71) of the EPM. These results suggest that the following analyses were not based on groups with distinctively different innately anxious responses to the conditions of the SAM apparatus.

### During-sam analysis of anxious behavior

**Figure 2:**
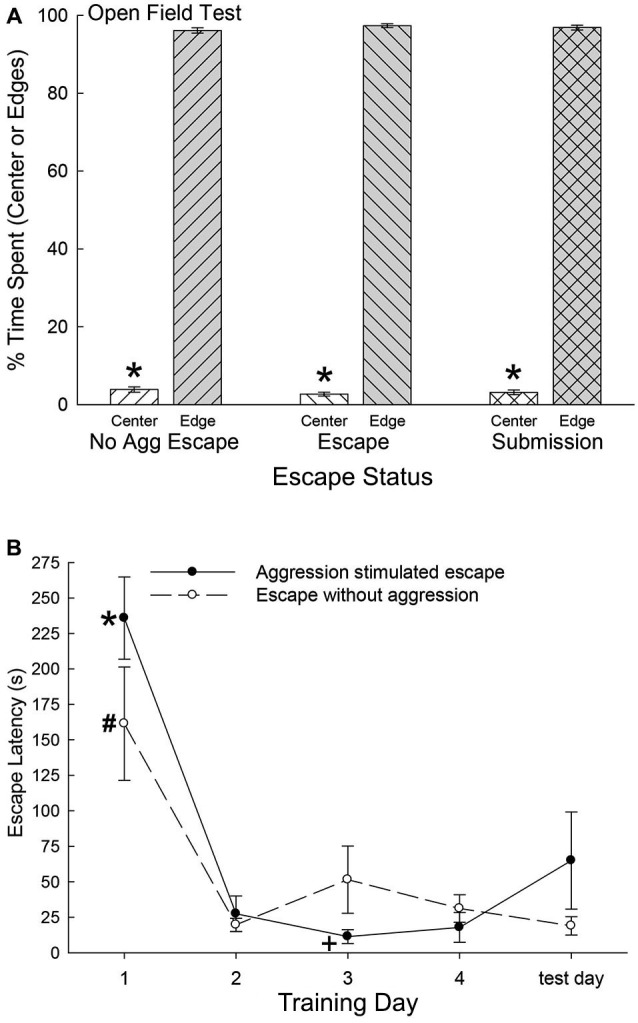
**(A) Similar open field anxiety. **(B)** Latency to escape diminishes quickly.** **(A)** Mean (± SEM) percent time spent in the open center (white bars) and edge (gray bars) portions of the open field interior arena of the SAM apparatus (* indicates statistical significance between center and edge preference, *p* < 0.05) for No Aggression (left hatching; *N* = 7), Escape (right hatching; *N* = 11) and Submission (cross hatching; *N* = 32) groups. **(B)** Mean (± SEM) latency to escape for the animals exposed to social aggression significantly (* indicates statistical significance *p* < 0.05; *N* = 11) decreased after training day 1 (∼236 s) and they continued to escape faster (+, under ∼45 s) for the duration of the experiment (solid line). Mice exposed to only the open field portion of the SAM apparatus followed a similar pattern of escape latency and continued to escape significantly (# *p* < 0.05) faster for the reminder of the experiment (dashed line).

As the SAM apparatus is essentially an open field, we used pre-escape behavior for both groups exhibiting escape behavior and non-social behaviors of submissive animals, to measure anxious behavior in an open field test. There were no significant differences among any groups on the time spent in the center of the open field (*F*_2,47_ = 0.36, *p* < 0.697; Figure 2A) or along the edges (*F*_2,40_ = 0.37, *p* < 0.69) of the open field portion of the SAM. However, each group exhibited extremely similar significant preference for the edge areas as compared with the center (No Aggression: *t*_6_ = 67.4, *p* < 0.001; Escape: *t*_10_ = 95.75, *p* < 0.001; Submissive: *t*_31_ = 77.8, *p* < 0.001; Figure 2B). These results suggest that the following analyses were not based on groups with distinctively different innately anxious responses to the conditions of the SAM apparatus.

### Latency to escape, submission, and fear conditioning

Mice that chose escape demonstrated a significantly (*F*_4,21_ = 30.18, *p* < 0.001; Figure 2B) decreased escape latency after training day 1 (∼236 s) and continued to escape significantly faster (under ∼45 s) for the duration of the experiment (Figure 2, solid line). Animals that remained submissively with a novel CD1, exhibited freezing behavior to the CS, and experienced prolonged social aggression for each training period (300 s). On test day (day 5) with CS alone (tone) and no US (novel CD1) mice that had previously chosen to utilize the escape route during training days 1 through 4 escaped from the open field portion of the SAM. Interestingly and importantly, submissive animals chose not to exploit the escape holes on test day, as they had not on training days. In contrast with submissive animals, the No Aggression group escaped on test day, and followed a similar significant (*F*_5,20_ = 11.51, *p* < 0.001; Figure 2) escape pattern as CD1-challenged escapers during training, with decreased escape latency after day 1 (Figure 2, dashed line).

**Figure 3:**
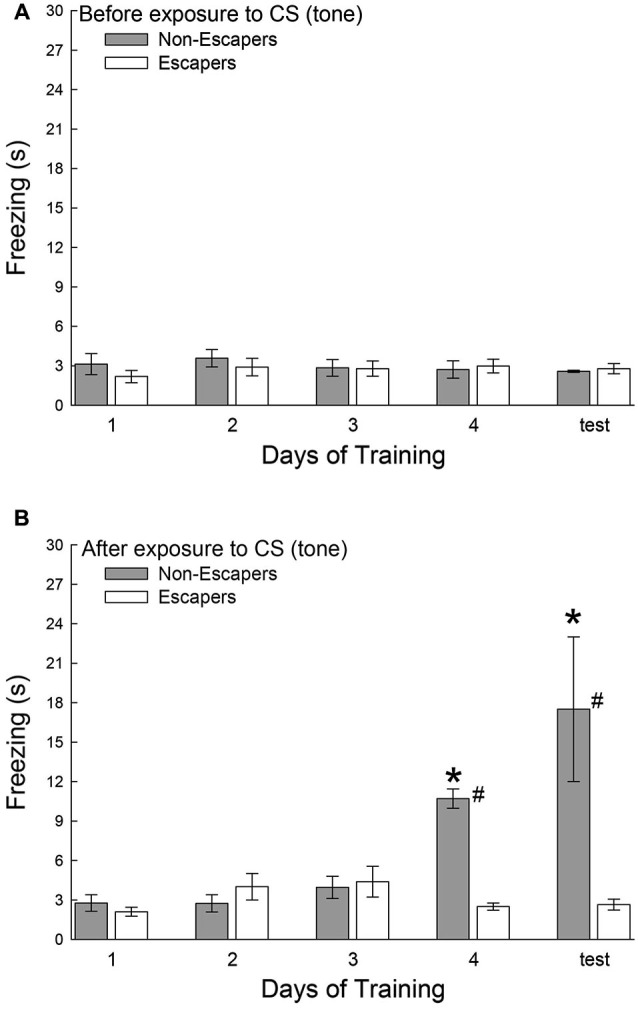
**Effects of decision-making on classical fear conditioning**. Mean (± SEM) duration of freezing **(A)** before and **(B)** after the conditioned stimulus (CS = tone), but prior to exposure to the aggressive CD1 and subsequent social interaction. Increased freezing was significant (* indicates statistical significance compared with days 1–3, *p* < 0.05; *N* = 5−9) only in mice that chose to remain submissively (Non-Escapers, gray bars) after training day 3. Escaping mice (white bars) do not exhibit Pavlovian conditioning to the auditory cue (CS = tone; # indicates significant differences between Non-Escapers and Escapers on that day, *p* < 0.05).

During each training session, and on test day, the C57BL6/N test mice were secluded for a 30 s period before a conditioned stimulus (CS = tone), and for an additional 30 s after the CS that preceded aggressive social interaction. During the period before the tone was presented, all groups exhibited very little freezing behavior, and there was no significant effect of training in Escapers (*F*_4,32_ = 0.31, *p* < 0.87; Figure 3A) or Non-Escapers (*F*_4,17_ = 0.32, *p* < 0.86; Figure 3A). After the CS, but during the 15 s of silence prior to agonistic interaction, test mice that escaped the aggression from the CD1 also exhibited very little freezing, for which there was no training effect (*F*_4,32_ = 1.83, *p* < 0.148; Figure 3B). In contrast, test mice that chose not to escape during aggressive encounters with a CD1 mouse exhibited a significant conditioned response by day 4 of training and on test day, (*F*_4,17_ = 10.37, *p* < 0.001; Figure 3B). This response was expressed in the form of increased freezing behavior following the tone and preceding the actual aggression.

During the period of seclusion before the social interaction, animals exhibited some escape behavior, putting their paws up on the cylinder wall, exploring for routes of egress. While this behavior did diminish over duration of the experiment, there was no evidence for classical conditioning in either group (*F*_1,140_ = 0.31, *p* < 0.578), no significant differences between groups (*F*_1,140_ = 0.05, *p* < 0.829), and no interaction of CS presentation by group (*F*_1,140_ = 1.87, *p* < 0.173).

### Plasma corticosterone

**Figure 4:**
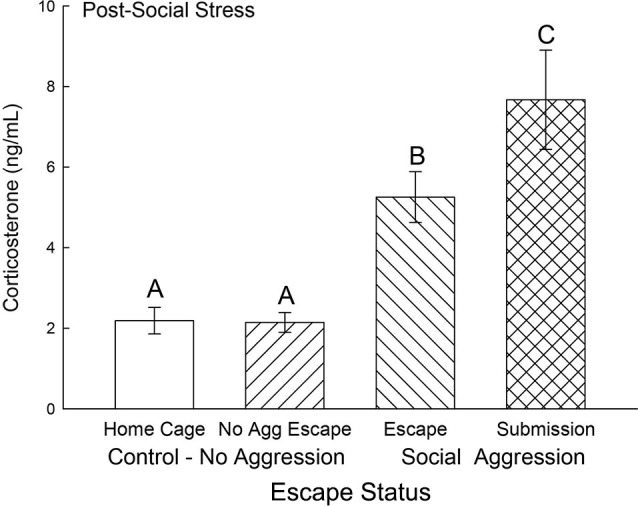
**Expectation of social aggression stimulates rapidly increased secretion of corticosterone**. Mean (± SEM) plasma corticosterone on test day (CS+, no US) for cage controls (clear bars; *N* = 12), after SAM exposure in mice not receiving aggression (bars with left hatch; *N* = 7), after SAM plus social interaction in mice that escaped aggression on days 1–4 (right hatch; *N* = 11), and submissive mice (cross hatch; *N* = 5). Letters above the bar (A, B, C) signify statistical significance (*p* < 0.05), such that bars that do not share a common letter (e.g., Escape = B vs. Submission = C) are significantly different, and those that do share a letter (e.g., Home Cage = A vs. No Aggression Escape = A) are not.

Plasma corticosterone concentrations on test day were typically low (near baseline at ∼2 ng/ml) and not significantly different for the two control groups, without treatment (Cage Control) and without aggression (No Aggression). However, both groups (Submission, Escape) that experienced social aggression had rapidly and significantly elevated (*F*_3,31_ = 16.85, *p* < 0.001; Figure 4) plasma corticosterone concentrations compared to both control groups. Plasma samples were taken 10 min after a 5 min final SAM trial following a CS (tone), but with no CD1 (US) present, and the group that did not choose escape (Submission) had significantly (*p* < 0.014) higher plasma corticosterone than those that did choose to use the escape route.

### Gene expression for BDNF and NPS

**Figure 5:**
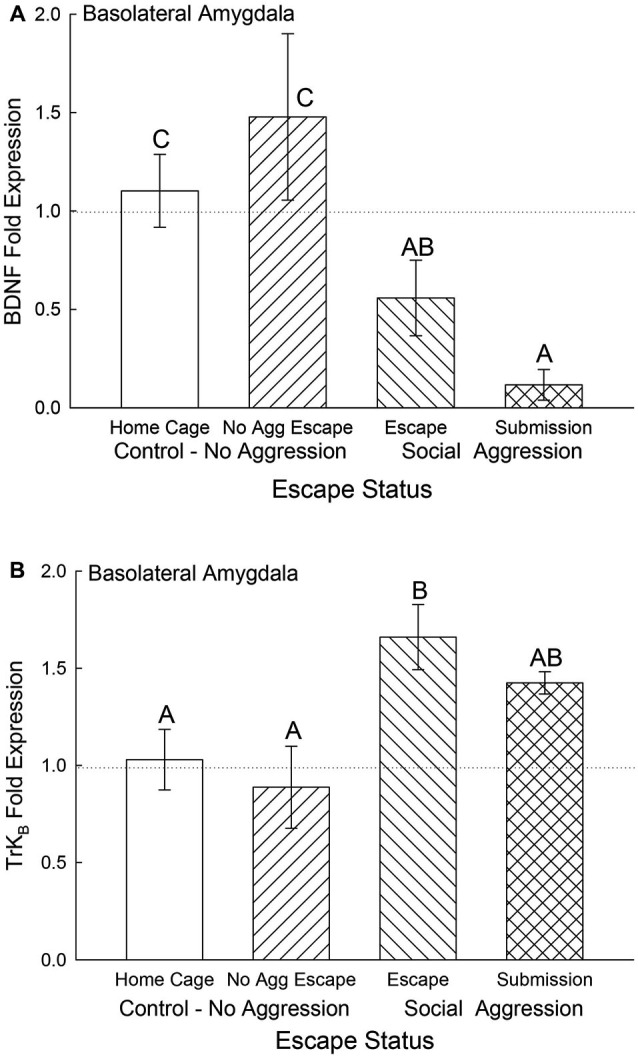
**Expectation of social aggression decreases BLA BDNF mRNA and modulates TrK_B_ mRNA**. Mean (± SEM) fold expression of **(A)** BDNF and **(B)** Trk_B_ mRNA from BLA on test day (CS+, US−) for cage controls (clear bars; BDNF *N* = 13; Trk_B_ *N* = 14), after SAM exposure in mice not receiving aggression (bars with left hatch; BDNF *N* = 7; Trk_B_ *N* = 6), after SAM plus social interaction in mice escaping aggression during training (right hatch; BDNF *N* = 7; Trk_B_ *N* = 7), and submissive mice (cross hatch; BDNF *N* = 4; Trk_B_ *N* = 4). Letters above the bar (A, B, C) signify statistical significance (*p* < 0.05), such that bars that do not share a common letter (e.g., BDNF Home Cage = C vs. Submission = A) are significantly different, and those that do share a letter (e.g., Trk_B_ Escape = B vs. Submission = AB) are not.

Expression of BDNF mRNA in the BLA was significantly (*F*_3,24_ = 4.5, *p* < 0.012; Figure 5A) decreased for animals exposed to aggression compared to No Aggression and Home Cage controls; whereas there was no significant difference between control groups. Submissive animals exhibited significantly (*p* < 0.026) reduced BDNF mRNA compared with controls but not significantly (*p* < 0.26) less than escapers. The reduction in BDNF gene expression for animals escaping aggression was at the *p* < 0.064 level compared to cage controls.

The BDNF receptor TrK_B_ mRNA gene expression in the BLA was significantly elevated by aggression-induced escape (*F*_3,27_ = 3.46, *p* < 0.03; Figure 5B). The BLA TrK_B_ expression in submissive animals was not significantly different from either controls or escapers.

**Figure 6:**
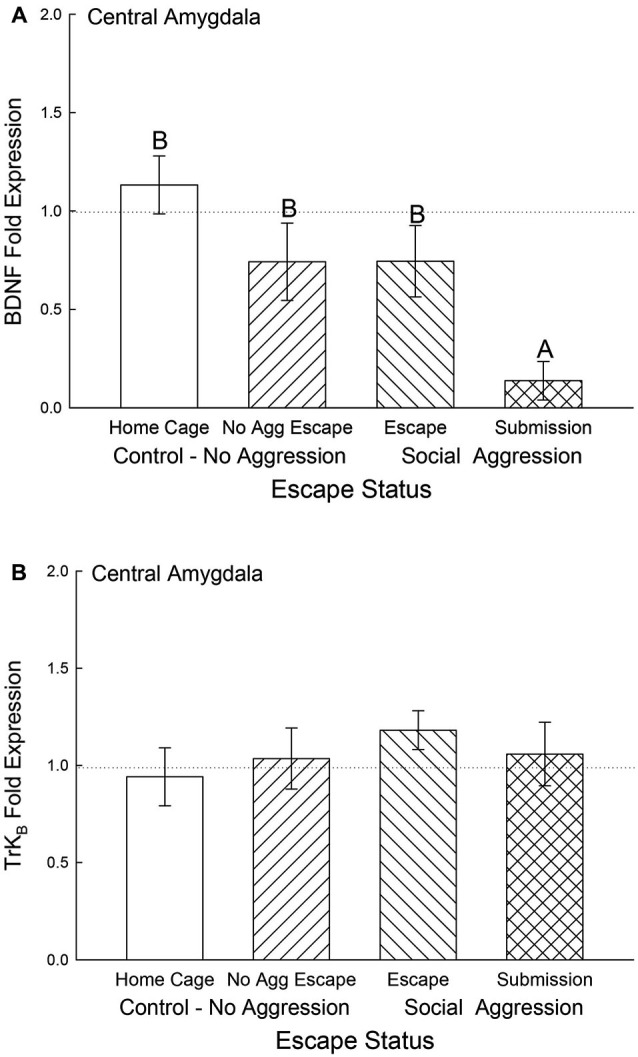
**Submissive mice exhibit decreased CeA BDNF mRNA**. Mean (± SEM) fold expression of **(A)** BDNF and **(B)** Trk_B_ mRNA from CeA on test day (CS+, US−) for cage controls (clear bars; BDNF *N* = 14; Trk_B_ *N* = 14), after SAM exposure in mice not receiving aggression (bars with left hatch; BDNF *N* = 6; Trk_B_ *N* = 6), after SAM plus social interaction in mice that escaped aggression during training (right hatch; BDNF *N* = 8; Trk_B_ *N* = 8) and mice that were submissive during training (cross hatch; BDNF *N* = 5; Trk_B_ *N* = 5). Letters above the bar (A, B) signify statistical significance (*p* < 0.05), such that bars that do not share a common letter (e.g., BDNF No Aggression Escape = B vs. Submission = A) are significantly different, and those that do share a letter (e.g., BDNF Home Cage = B vs. Escape = B) or have no letters at all are not.

In the CeA, BDNF mRNA gene expression was not significantly different among control and escape groups, but it was significantly (*F*_3,29_ = 5.12, *p* < 0.006) decreased for the submissive mice (*p* < 0.003; Figure 6A). There were no significant differences for TrK_B_ mRNA gene expression in the CeA (*F*_3,29_ = 0.49, *p* < 0.695; Figure 6B).

**Figure 7:**
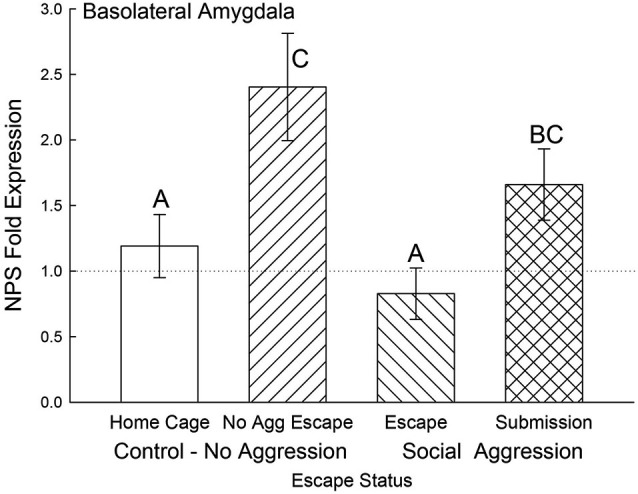
**Open field exposure and expectation of submission to social aggression elevate BLA NPS expression**. Mean (± SEM) fold expression of NPS mRNA in BLA on test day (CS+, US−) for cage controls (clear bars; *N* = 11), after SAM exposure in mice not receiving aggression (bars with left hatch; *N* = 4), after SAM plus social interaction in mice escaping aggression (right hatch; *N* = 5) and submissive mice (cross hatch; *N* = 4). Letters above the bar (A, B, C) signify statistical significance (*p* < 0.05), such that bars that do not share a common letter (e.g., Escape = A vs. Submission = BC) are significantly different, and those that do share a letter (e.g., No Aggression Escape = C vs. Submission = BC) are not.

In the BLA, NPS mRNA was significantly elevated for the submissive animals and No Aggression controls (*F*_3,20_ = 4.25, *p* < 0.018) compared to the Escape group and Cage Controls. Gene expression for BLA NPS was not significantly lower for submissive animals compared to the No Aggression escape group (Figure 7).

**Figure 8:**
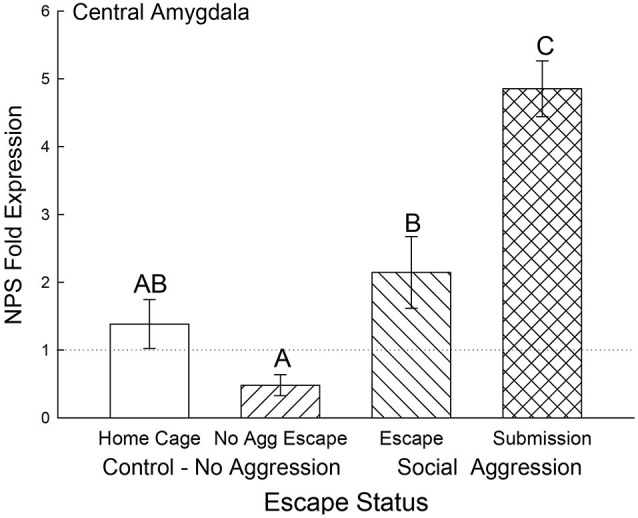
**Expectation of social aggression elevates central amygdala NPS expression**. Mean (± SEM) fold expression of NPS mRNA in CeA on test day (CS+, US−) after SAM social interaction for cage control (clear bars; *N* = 11), after SAM exposure in mice not receiving aggression (bars with left hatch; *N* = 6), after SAM plus social interaction in mice escaping aggression (right hatch; *N* = 7) and submissive mice (cross hatch; *N* = 4). Letters above the bar (A, B, C) signify statistical significance (*p* < 0.05), such that bars that do not share a common letter (e.g., Escape = B vs. Submission = C) are significantly different, and those that do share a letter (e.g., Home Cage = AB vs. No Aggression Escape = A) are not.

In the CeA, NPS gene expression was elevated for animals that received social aggression compared to controls (*F*_3,24_ = 14.04, *p* < 0.001; Figure 8), with the exception that the Escape group was not significantly different from Cage Controls. Interestingly, the Submissive group had significantly (*p* < 0.001) elevated NPS mRNA in the CeA when compared to animals escaping social aggression (Figure 8).

Other genes of interest in the BLA and CeA that were tested for all groups but resulted in no significant difference from controls were NPY, GluR_1_ and GluR_4_. In the BLA, NPY (*F*_2,21_ = 1.4, *p* < 0.25; data not shown), GluR_1_ (*F*_2,19_ = 1.42, *p* < 0.27), and GluR_4_ (*F*_2,20_ = 0.6, *p* < 0.59) were not significantly affected by aggression or escape, as measured on the test day after 4 days of training. Similarly in the CeA, aggression and/or escape had no effect on NPY (*F*_2,24_ = 1.1, *p* < 0.35; data not shown), GluR_1_ (*F*_2,24_ = 1.5, *p* < 0.25), and GluR_4_ (*F*_2,20_ = 3.0, *p* < 0.075) expression on test day when presented with a tone.

**Figure 9:**
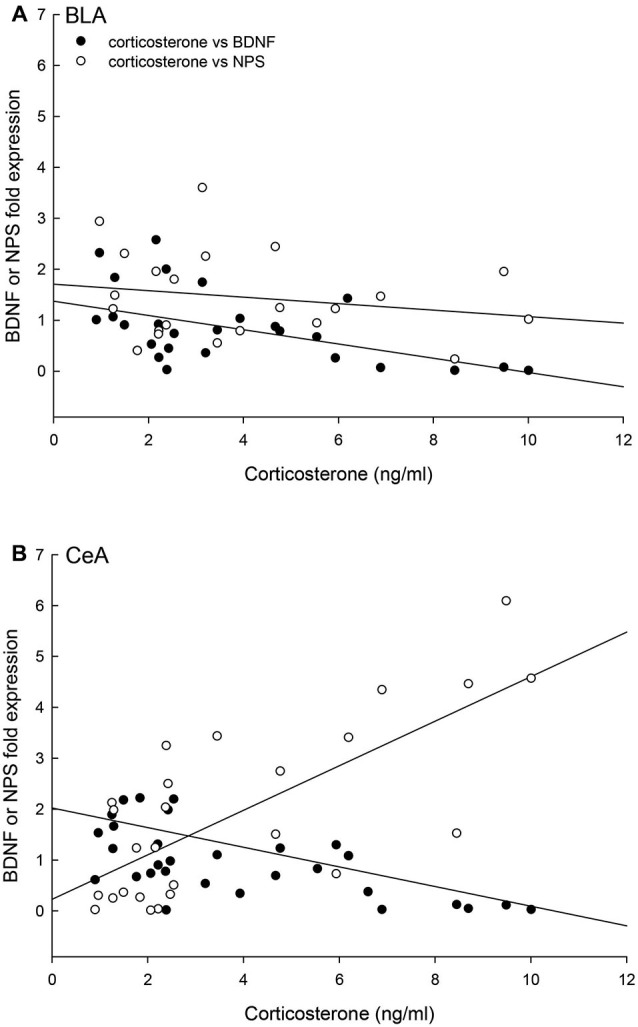
**Concentrations of corticosterone, BDNF and NPS gene expression are correlated. (A)** In the BLA, the individual (Control *N* = 14; No Aggression *N* = 7; Escape *N* = 8; No Escape *N* = 8) corticosterone, BDNF mRNA, and NPS mRNA concentrations are correlated (multiple regression analysis *r*^2^ = 0.43, *p* < 0.005; 3D regression analysis *r*^2^ = 0.20, *p* < 0.12); with a negative regression for BDNF and corticosterone (*p* < 0.002), no statistical relationship between NPS and corticosterone (*p* < 0.05), and a trend toward a positive correlation between BDNF and NPS fold expression (*p* < 0.098). **(B)** In the CeA, the individual (Control *N* = 14; No Aggression *N* = 7; Escape *N* = 8; No Escape *N* = 5) corticosterone, BDNF mRNA, and NPS mRNA concentrations are correlated in a three-dimensional relationship (multiple regression analysis *r*^2^ = 0.62, *p* < 0.001; 3D regression analysis *r*^2^ = 0.54, *p* < 0.0002); with negative regressions for BDNF and corticosterone (*p* < 0.043) and BDNF and NPS (*p* < 0.016) and a positive correlation between corticosterone and NPS fold expression (*p* < 0.002).

Finally, the relationships of individual plasma corticosterone concentrations with BDNF and NPS gene expression were significantly correlated for the BLA (multiple regression: *F*_2,21_ = 7.15, *p* < 0.005, *r*^2^ = 0.43; 3D regression: *F*_2,24_ = 13.13, *p* < 0.0002, *r*^2^ = 0.54) and the CeA (multiple regression: *F*_2,24_ = 17.67, *p* < 0.001, *r*^2^ = 0.62; 3D regression: *F*_2,24_ = 13.13, *p* < 0.0002, *r*^2^ = 0.54; Figure 9). In the CeA, the relationship between corticosterone and BDNF expression is the opposite, with contrasting regression slopes, of the relationship between corticosterone and NPS expression (Figure 9).

## Discussion

Changes in behavior can reflect a decision-making process that includes both executive and emotional circuits (amygdala, ventral and dorsal striatum, anterior cingulate, orbitofrontal cortex and dorsolateral PFC) in the brain (de Visser et al., 2011a,b). While we cannot be sure that the decision-making process we describe includes an active and explicit consideration of the alternatives by test mice, these self-selected choices in escape and submissive behavior appear to be more than simply innate, for the following reasons. First, while populations of C57BL6/N mice have been demonstrated to show significant variability with respect to behaviors associated with affective disorders (Arendt et al., 2013), we found no evidence of innately anxious behavior by pre-testing on the EPM, and during testing in the open field internal arena of the SAM, in self-selected escaping and submissive groups, or controls. Second and surprisingly, about half of the animals that experienced severe social aggression chose to remain submissively, despite unremitting attacks from a novel CD1 and the anxiety associated with the open field portion of the SAM. However, in this cohort approximately 7% of the submissive animals began by escaping from the behavioral arena. Clearly the test mice are capable of both escape and submissive behavior, and over time choose a stable submissive behavioral response. Although both escape and submission are plausible alternatives for each individual, the choice of one clearly includes an emotional imperative, as we hypothesized. Third, while both social experienced groups clearly demonstrate learning, only submissive animals exhibit classical conditioning (Carpenter and Summers, 2009). Finally, while mice prefer to interact with other mice rather than exploring novel objects (such as the escape route; File and Hyde, 1978), half the animals in our experiments chose to escape. Again, it is important to note that approximately 9% of animals that eventually demonstrated a stable escape strategy began by remaining in the arena and showing submissive behavior during the initial interactions.

While social defeat was clearly a motivation for escape in other species (trout, rats, hamsters) (Carpenter and Summers, 2009; Arendt et al., 2012), animals that did not experience social defeat also chose egress from the behavioral area. This suggests that escape was preferable to the anxiety of the open field (Smagin et al., 1996; Ramos et al., 1997; Park et al., 2001; Takahashi et al., 2001). While anxiety associated with the exposed portions of an open field is well-documented (Ramos et al., 1997; Park et al., 2001; Takahashi et al., 2001), and verified in the internal open field arena in our experiments (Figure 2A), the SAM behavioral apparatus demonstrates this effect in control animals (no social aggression) with an active response and end point. Again, it was surprising that the latency to escape was similar regardless of the presence of intense and sustained social antagonism. The escape route was used to alleviate both exposure anxiety and social defeat, but the combination of these two stressors didn’t change the time necessary to find the hole, decide to use it, and to escape. Behavioral analysis indicated that visual identification of the hole occurred in the initial moments of the first training interaction, and traversing the escape route takes less than 2 s. This suggests that it is the decision-making process that primarily determines the latency to escape for both groups regardless of the presence of aggression. In rainbow trout, the duration of the decision-making process was determined by social interaction, specifically the timing of escape was controlled by the inattentiveness of the aggressor (Carpenter and Summers, 2009; Summers et al., 2011).

The experience of the SAM produces dramatically differential behavioral outputs from a relatively homogeneous population of laboratory mice, but also results in distinctly different learning mechanisms to modify and maintain ongoing behavioral phenotypes (Carpenter and Summers, 2009). Animals that learned to escape did so quickly (Figure 2). By day 2, Escapers eluded further aggressive insult in about ^1^/_8_^th^ the time it took on day 1. In contrast, animals that remained submissively, clearly demonstrated classical fear conditioning to an auditory stimulus presented prior to social aggression. In this case, the learning effect (increased freezing) was not evident until day 4 (Figure 3). Therefore both the mode and timing of social learning was distinctly different between animals utilizing these self-selected adaptive responses. In addition, our results suggest that using the escape route significantly alleviated stress (Figure 4).

The post-training elevation in corticosterone to the CS is also suggestive of a Pavlovian stress response that was previously demonstrated for rainbow trout in a similar model (Carpenter and Summers, 2009). For mice receiving aggressive social interactions plasma corticosterone concentrations were significantly elevated regardless of their proclivity to use the escape hole. However even though both submissive and escaping animals express this rapid, early stress response, escaping animals have a significantly smaller increase in corticosterone. This suggests that escaping social aggression ameliorates stress. Had there been no potential for escape via the hole, we would have expected that the open field alone would have stimulated corticosterone secretion in No Aggression controls (Arendt et al., 2012). Elevated corticosterone has been demonstrated to have very rapid effects on neurotransmitter and behavioral responses (Summers et al., 2003, 2005b), which suggests the potential for influencing the decision-making process (Graham et al., 2010; Gourley et al., 2012; Shafiei et al., 2012; Koot et al., 2013).

The neurotrophic factor BDNF in the amygdala is associated with fear learning (Orsini and Maren, 2012). Expression of BDNF mRNA and phosphorylation of its TrK_B_ receptor in the BLA are increased in response to Pavlovian aversive learning (Rattiner et al., 2004). Antagonism of the TrK_B_ receptor activity with a dominant-negative isoform blocked fear acquisition. We measured BDNF and TrK_B_ gene expression 4 days after the initial acquisition of stress-stimulated escape behavior, and BLA as well as CeA BDNF mRNA levels were significantly depressed in socially submissive mice. On test day, there was a significant stress-induced elevation in plasma corticosterone which has been demonstrated to reduce BDNF expression in hippocampus (Pizarro et al., 2004) PFC (Gourley et al., 2012) and BLA (Pizarro et al., 2004). However it appears that in submissive animals the expression of BDNF mRNA was distinctly inhibited, which may suggest that with prolonged social stress BDNF is no longer required in the amygdala for expression of a normal fear response. It is possible that the decrease in BDNF for submissive animals is a result of classical fear conditioning.

Any behavioral coping strategy must include a mechanism for reducing anxiety, which led us to investigate NPS expression in the amygdala. While the majority of NPS is produced in the pericerulear region of the brainstem, a very small number of NPS cells with precursor mRNA signals have been found in the amygdala (Xu et al., 2007; Guerrini et al., 2010; Deglincerti and Jaffrey, 2012; Jung et al., 2012). In addition, mRNA expression, including that of secreted peptides, has been demonstrated in distal neuronal rami, including dendrites, axons and terminals (Alvarez et al., 2000; Piper and Holt, 2004), with translation stimulated by neuronal activity (Buxbaum et al., 2014; Park et al., 2014). The NPS receptor has been located throughout the amygdaloid complex, with heavy expression in the intercalated (ITC) cells between the BLA and CeA but also expression in the BLA and CeA as well (Xu et al., 2007). The NPS peptide has been demonstrated to modulate the local circuitry leading to and from the BLA which results in control of fear expression and fear potentiated startle, regulation of anxiety, depression, and panic disorder, as well as promoting memory consolidation (Xu et al., 2004; Jungling et al., 2008; Meis et al., 2008; Duangdao et al., 2009; Fendt et al., 2010; Pape et al., 2010; Dannlowski et al., 2011; Ebner et al., 2011; Okamura et al., 2011; Enquist et al., 2012). Our results suggest that NPS gene expression exists in the regions in or near the BLA and CeA and that mRNA levels increase in response to continuous social submission, and as such may be a part of an insufficient compensatory mechanism. However, NPS gene expression in these regions is highly context specific and is enhanced in response to escape from open field exposure alone (in BLA for No Aggression controls) and by self-selected submission to social defeat (in BLA and CeA), but remains unaffected in the BLA of animals escaping social defeat. In animals escaping social aggression, the egress behavior is exactly the same as that for animals escaping the open field alone (in the absence of aggression). They use the same escape route, the exposure to the open field is the same, but social interaction with a conspecific changes the context entirely; and the NPS response is also changed. In the CeA (may include some of the median paracapsular ITC cells adjacent to the CeA), the story of NPS expression is significantly simpler than for the BLA. Only aggression enhances NPS mRNA expression and it is significantly greater in socially defeated submissive animals. Taken together the data suggest that NPS expression in or near the BLA or CeA increases in response to social and environmental stressors. The relationship between BLA and CeA NPS expression may involve contextual learning, which potentially would include the recently hypothesized NPS responsive circuitry that is thought to shape amygdalar activity via input from the endopiriform cortex (Jungling et al., 2008; Meis et al., 2008; Pape et al., 2010).

The combined results for plasma corticosterone, BDNF and NPS gene expression have implications for understanding fear learning that arise from contextual differences during environmentally and socially stressful conditions. As has previously been demonstrated, experiencing social aggression followed by an increase in plasma corticosterone decreases BDNF gene expression in the CeA and BLA (Pizarro et al., 2004). Our results suggest that this corticosteroid inhibition occurs in a dose-dependent manner (Figures 4, 9). In the fear-learning and fear-expression regions of the amygdala, the BLA and CeA respectively, inhibition of BDNF expression may depend on social stress-stimulated elevation of both NPS expression and corticosterone concentrations, as suggested by the significant multiple regression analyses (Figure 9). Results from a three-dimensional regression suggest a speculative conclusion, which requires further experimentation. If correct, NPS may play an important role in contextually derived adaptive behavior. In the CeA, this appears to be a straightforward relationship; when plasma corticosterone concentrations are high, and BDNF expression is low, NPS gene expression is also high. In the BLA however, mice choosing escape from exposure without any aggressive social stress, have elevated NPS expression but low corticosterone, and normal BDNF gene expression. It may be that only when NPS expression and corticosterone concentrations are both elevated is BDNF expression fully inhibited. Additionally, pharmacological therapeutics using NPS have previously been shown to be effective intranasally (Ionescu et al., 2012), and have the potential for applications that are contextually nuanced.

In conclusion, the newly developed SAM provides a way of testing exposure to an open field with an active response (escaping the open field). In addition, it is possible to discern a significant and measurable decision-making period between recognition of the escape hole and utilizing the escape route. Our model, which includes an auditory CS and a social aggressor as a US, provides a non-stochastic decision-making process that exposes early behavioral plasticity and an eventual stable strategy for social interaction or escape, rather than exploiting pre-existing phenotypes for anxious behavior. Classic open field analysis of the non-social behavior in the SAM interior arena indicates that decisions to escape or remain submissively were not influenced by innately anxious proclivities. The SAM produces a non-intuitive result: submissive animals remain exposed in the open field while oppressed by social aggression rather than escape. In the absence of aggression escape from the open field is the natural response. Submissive animals (Non-Escapers) are not predisposed to an anxious phenotype but demonstrate classical fear conditioning to an auditory stimulus after 4 days of training whereas escaping animals do not. Escaping from social aggression provides a decrease in the HPA stress response and no change in BLA NPS gene expression with a limited reduction in BLA BDNF gene expression. Submissive animals on the other hand, show rapidly and substantially elevated plasma corticosterone that coincides with elevated NPS and progressively diminished BDNF gene expression in both BLA and CeA, which may be influenced by fear conditioning. A three-dimensional regression suggests a contextually linked interwoven relationship between amygdalar BDNF, NPS and plasma corticosterone. The context of our research thus far has been anxious behavior, however other psychiatric disorders are highly comorbid with anxiety, and a current limitation of the SAM is that it remains to be validated with standard behavioral tests for depression, learning, and conditioned fear. Another limitation of the SAM is that we are currently unable to discern individual proactive and reactive phenotypes (Koolhaas et al., 2010). In addition, the SAM has not been measured against more stochastic decision-making tests. While these comparisons are necessary, a distinctive value of our model is that it exploits naturally occurring rodent behavior, in contrast with tests artificially constructed to mimic human psychological disorders but not congruent with evolutionarily adaptive behaviors natural to rodents (Blanchard et al., 2013). While the decision-making that takes place in SAM experiments is simple, the results from these experiments reveal complicated social- and context-dependent neural changes that likely also underlie domestically abusive situations, bullying, job-related stress, post-traumatic stress disorder, addiction, anxiety, and depression. All of these conditions share symptomologies that are aggravated by stressful conditions, and in which adaptive decision-making is compromised.

## Conflict of interest statement

The authors declare that the research was conducted in the absence of any commercial or financial relationships that could be construed as a potential conflict of interest.
